# Synthesis of a New *ent*-Cyclozonarone Angular Analog, and Comparison of Its Cytotoxicity and Apoptotic Effects with *ent*-Cyclozonarone

**DOI:** 10.3390/molecules18055517

**Published:** 2013-05-13

**Authors:** Natalia Quiñones Sobarzo, Iván Montenegro Venegas, Cristian Salas Sánchez, Luis Espinoza Catalán, Cristóbal Carrasco Rojas, Valentina Ulloa Valdivia, Joan Villena García, Mauricio Cuellar Fritis

**Affiliations:** 1 Facultad de Farmacia, Universidad de Valparaíso, Av. Gran Bretaña N° 1093, Valparaíso, Chile; 2 Facultad de Química, Pontificia Universidad Católica de Chile, Vicuña Mackenna N° 4860, Santiago, Chile; 3 Departamento de Química, Universidad Técnica Federico Santa María, Av. España N° 1680, Valparaíso, Chile; 4 Centro de Investigaciones Biomédicas, Escuela de Medicina, Universidad de Valparaíso, Av. Hontaneda N° 2664, Valparaíso, Chile

**Keywords:** sesquiterpenequinone, *ent*-cyclozonarone, cancer cells, cytotoxicity, apoptosis, caspase 3, mitochondrial membrane permeability

## Abstract

The synthesis of a newangular analog **11** of cyclozonarone was achieved via Diels-Alder reaction between a sesquiterpene-1,3-diene and 1,4-benzoquinone. The cytotoxic activity of *ent*-cyclozonarone [(+)-**10**] and the angular (−)-cyclozonarone analog **11** has been determined in three human cancer cell lines and in normal fibroblasts using the sulforhodamine B assay. The analyzed isomers induce cell death in different cancer cell lines by eliciting nuclear condensation and fragmentation, decreasing mitochondrial membrane permeability and increasing caspase-3 activity, all traits indicating apoptosis, with the effects of (+)-**10** being stronger than those of **11** in all cases.

## 1. Introduction

**Figure 1:**
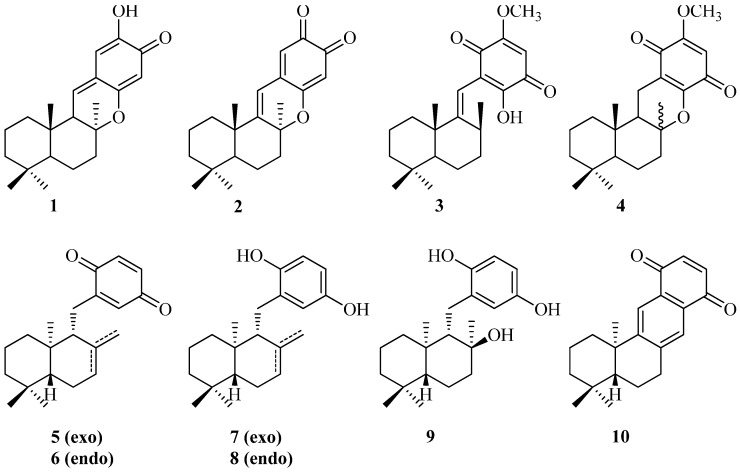
Some examples of natural sesquiterpenequinones.

Sesquiterpene-substituted quinones and related molecules constitute an important class of bioactive marine natural products [1], which includes the cytotoxic, antiviral and antifungal sponge metabolites puupehenone (**1**) and puupehedione (**2**), isolated from sponges, mainly of the orders *Verongida* and *Dictyoceratida* [2], and the sesquiterpene quinones spongiaquinone (**3**) and cyclospongiaquinone-1 (**4**), isolated from *Stelospongia conulata* [3,4] (Figure 1). On the other hand, the sesquiterpene quinines hydroquinones, zonarone (**5**), isozonarone (**6**), zonarol (**7**), isozonarol (**8**), yahazunol (**9**) and (−)-cyclozonarone (**10**) have been isolated from the brown alga *Dictyopteris undulata* [5] (Figure 1). Natural(−)-cyclozonarone (**10**) possesses powerful feeding-deterrent activity towards young abalones and furthermore it shows anti-cancer activity [5,6]. On the other hand, (+)-cyclozonarone shows antileshmanial activity and was recently shown to be cytotoxic toward different cancer cell lines [7,8]. The absolute configuration of **10** was established through a six-step synthesis route, starting from natural (−)polygodial, leading us to the synthetic enantiomer (+)-cyclozonarone [9]. Later, (−)-cyclozonarone was synthesized starting from (+)-albicanol [10]. Both synthetic routes were based on the Diels-Alder reaction.

In the literaturequinone/hydroquinone cytotoxicity is usually attributed to two processes: redox cycling of quinones, resulting in the generation of reactive oxygen species which can damage biomolecules, and inhibition of mitochondrial function and electrophilic arylation of critical cellular nucleophiles. Both mechanisms can result in oxidative stress and cell death [11,12].

Apoptosis or programmed cell death not only plays a crucial role in a range of pathological conditions, but is now recognized as an important component of multi-step carcinogenesis [13,14]. This physiological phenomenon represents terminal morphological and biochemical events and occurs through the activation of a cell-intrinsic suicide program [15]. This program is carried out following internal, as well as external signals, and is divided into various phases terminating with signals that initiate chromatin condensation, characterized by DNA fragmentation as well as by the loss of mitochondrial membrane integrity and release of molecules that initiate the activation of intracellular protease [14,16,17]. Mitochondria play a crucial role in the apoptotic cascade by serving as a center where apoptotic signals converge [18], given that changes induced in the mitochondrial membrane potential (MMP) have been reported previously to represent a determining step in the execution of cell death [19,20]. Reduction of the mitochondrial membrane potential causes the opening of the mitochondrial permeability transition pore, which leads to the release of apoptogenic factors and activation of caspases that are responsible for the morphological and biochemical events that characterize classical apoptosis [21,22].

**Scheme 1:**
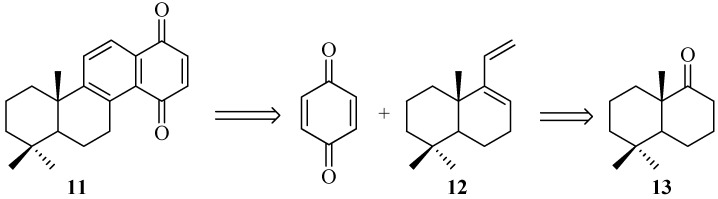
Retrosynthetic analysis to obtainof (+)-cyclozonarone.

Due to the interest of these meroterpenoids and other related compounds several synthetic strategies have been reported [23], and specifically we decided to synthesize an angular isomer **11** of (+)-cyclozonarone, easily prepared in a single step by a Diels-Alder reaction of 1,3 diene **12**, prepared in turn from ketone **13**, and 1,4-benzoquinone (Scheme 1).

In addition, we have evaluated both sesquiterpene quinones **10** and **11** for their*invitro* cytotoxicity against cultured human colon and prostate cancer cells and human dermal fibroblasts (DHF). Moreover, in order to determine if they act as apoptosis inducers, we evaluated the condensation and fragmentation of chromatin determined by Hoechst staining, the activity of caspase 3 and the permeabilization of the mitochondrial membrane by rhodamine 123 staining and the activity of caspase 3 [24,25].

## 2. Results and Discussion

### 2.1. Chemistry

When polygodial (**14**), obtained from the hexane extract of the bark of *Drimyswinteri*Forst. [7], was treated with sodium borohydride in methanol at room temperature it produced diol **15** in excellent yield. It was previously reported that treatment of diol **15** with one equivalent of *tert*-butyl-diphenylsilyl chloride in DMF, in the presence of imidazole, produced silyl ether **16**, with specificity towards the allylic alcohol, and excellent yield [26]. Unsaturated alcohol **16** was subjected to oxidation with Jones reagent affording enone **17** in moderate yield. Deprotection of silylether **17** with tetrabutylammonium fluoride in anhydrous tetrahydrofuran furnished alcohol **18** quantitatively. Catalytic hydrogenation of **18** afforded a mixture **19** of epimeric diols which, treated with Jones reagent, afforded the unsaturated ketone **13**, that had been obtained previously from confertifoline [27,28].

**Scheme 2:**
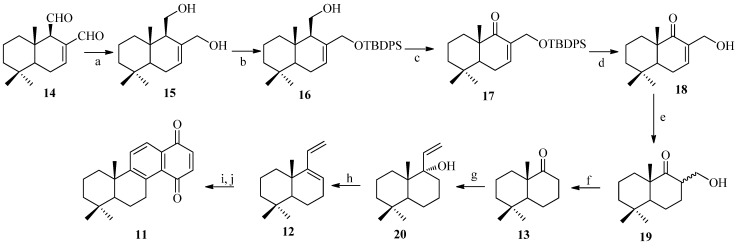
Synthesis of compound **11**.

Diene **12** was obtained by dehydration of allyl alcohol **20**, prepared by addition of vinylmagnesium bromide to ketone **13**. Finally, according to Scheme 2, diene **12** was condensed with 1,4-benzoquinone in benzene under thermal activation, and treated subsequently with DBU giving access to the aromatized product **11** in 33% yield.

The structural confirmation of new compound **11** was based on a careful analysis of its ^1^H- and ^13^C-NMR spectra, with the help of a combination of 1D and 2D experiments, particularlyheteronuclear multiple-bond correlations (HMBC and HSQC).

**Figure 2:**
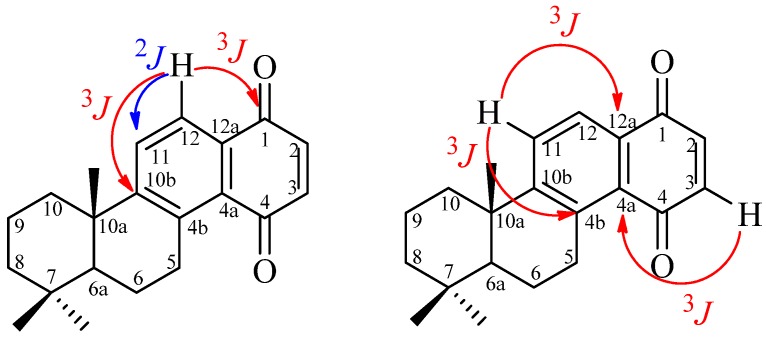
Major HMBC correlations observed for compound **11**.

In the ^1^H-NMR spectrum of **11**, the signals at δ = 7.98 ppm (1H, d, *J* = 8.3Hz) and δ = 7.72 ppm (1H, d, *J* = 8.3Hz) were to assigned to the H-12 and H-11 hydrogens, respectively. The signal at δ = 7.98 ppm showed an HSQC ^1^*J*_HC_ correlation with C-12 at δ_C_ = 124.9 ppm and an HMBC ^3^*J*_HC_ correlation with carbonyl carbon C-1 at δ_C_ = 185.6 ppm. Additionally, H-12 showed HMBC correlations with the quaternary aromatic carbon C-10b (δ_C_ = 158.3 ppm; ^3^*J*_HC_) and with C-11 (δ_C_ = 129.2 ppm; ^2^*J*_HC_) (see Figure 2).

On the other hand, the signal at δ_H_ = 7.72 ppm (H-11) showed an HSQC correlation with C-11 at δ_C_ = 124.9 ppm (^1^*J*_HC_) and HMBC correlations with C-12a (δ_C_ = 131.3 ppm) and C-4b (δ_C_ = 139.9 ppm; ^3^*J*_HC_). Finally,the carbonyl carbons C-1 (δ_C_ = 185.6 ppm) and C-4 (δ_C_ = 187.7 ppm) were unequivocally assigned when H-12 showed an HMBC ^3^*J*_HC_ correlation with C-1. Additionally, in the ^13^C-NMR spectrum the signal at δ_C_ = 130.2 ppm was assigned to C-4a by observation of HMBC ^3^*J*_HC_ correlations with H-2 and/or H-3 at δ = 6.87 ppm (s, 2H) All these correlations are shown in Figure 2.

### 2.2. Biology

The cytotoxicity of the compounds was evaluated *in vitro* against different cancer cell lines: HT29 colon cancer, PC-3 and DU-145 human prostate cancer and one non-tumoral cell line, human dermal fibroblasts (DHF). A conventional colorimetric assay was set up to estimate the IC_50_ values, which represent the concentration of a drug that is required for 50% inhibition *in vitro* after 72 h of continuous exposure to the test compounds. Four serial dilutions (from 12.5 to 100 µM) for each sample were evaluated in triplicate. The results obtained from these assays are shown in Table 1. The highest cytotoxicity values were observed for *ent*-cyclozonarone[(+)-(**10**)] in all cell lines tested and were approximately twice more active than those of compound **11**. The cytotoxicity of compounds (+)-**10** and **11** in fibroblasts is lower than in the cancer cell lines under study, indicating that the compounds are selectively toxic to cancer cells.

**Figure 3:**
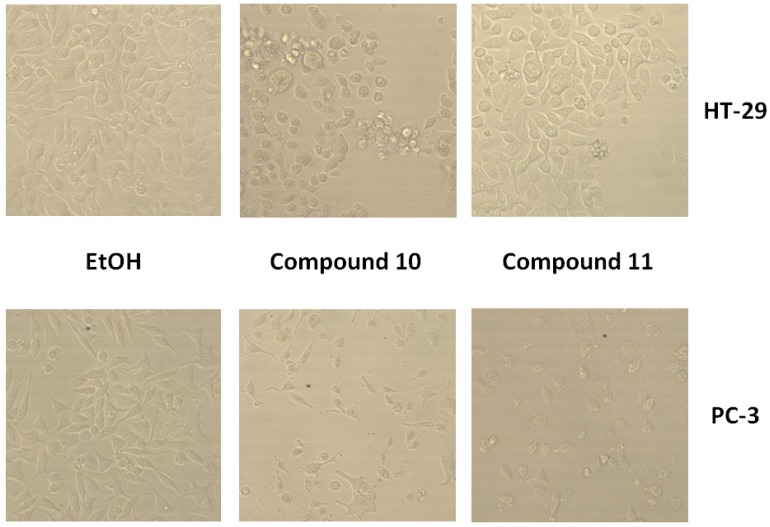
Effect of tested compounds on the morphology of HT-29 and PC-3 cells. Images obtained with an inverted phase contrast microscope (200×) after 24 h exposure of the cells to 25 μM (+)-**10** or **11**.

Since compounds (+)-**10** and **11** had strong inhibitory effects on the growth of the cancer cell types tested, we decided to study the effect of these compounds in greater detail. First, we analyzed the appearance of morphological changes in the cells treated with 25 μM compound for 24 h. Direct observation using a phase contrast microscope revealed that the morphology of PC-3 and HT-29 cells was severely distorted and cells became rounded, more clearly after treatment with (+)-**10**. Moreover, the cells showed a reduction in number, indicating an increasing progression toward cell death. The control-treated cells displayed normal and healthy shapes (Figure 3).

**Figure 4:**
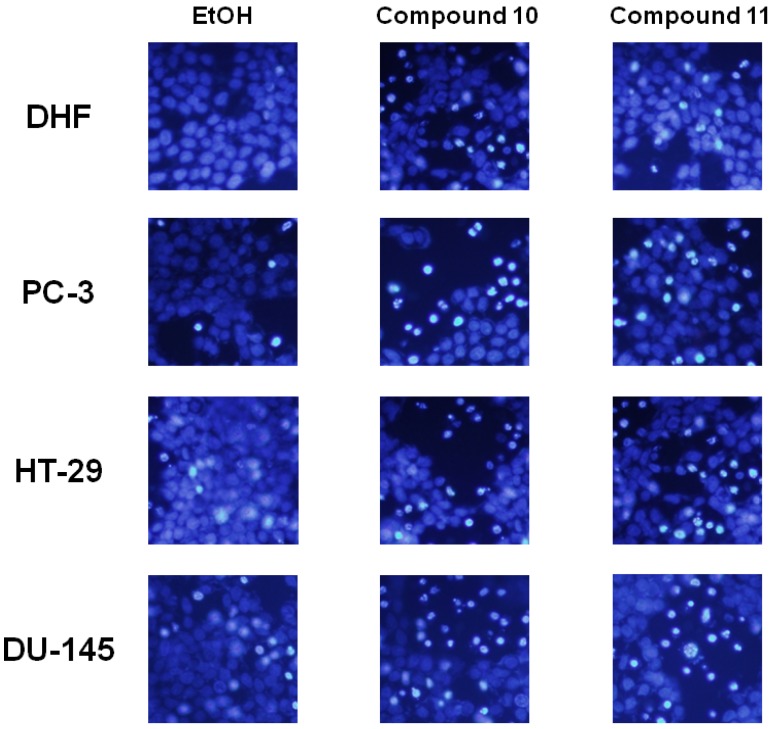
Effect of *ent*-cyclozonarone and compound 11 on chromatin condensation and fragmentation. PC-3, HT-29, DU-145 and DHF cells treated with either compound were stained with Ho33342 (400×). Representative photographs of nuclear morphologic changes observed by fluorescent microscopy of the treated cells.

To elucidate whether compounds (+)-**10** and **11** reduced cell viability of HT29, PC-3, DU-145 and DHF cells by inducing apoptosis, cells treated with each compound were examined after Hoechst 33342 staining. The nuclear changes in HT29, PC-3, DU-145 and HDF cells were also observed under a fluorescence microscope (200×). Exposure to compounds (+)-**10** and **11** significantly affected the condensation and fragmentation of the nuclei in the treated cells (Figure 4). Following incubation with the compounds for 24 h, the fluorescent emission was denser and brighter compared to control-treated cells. Also, the cells showed chromatin condensation and karyopyknosis, which are typical apoptotic phenomena.

**Figure 5:**
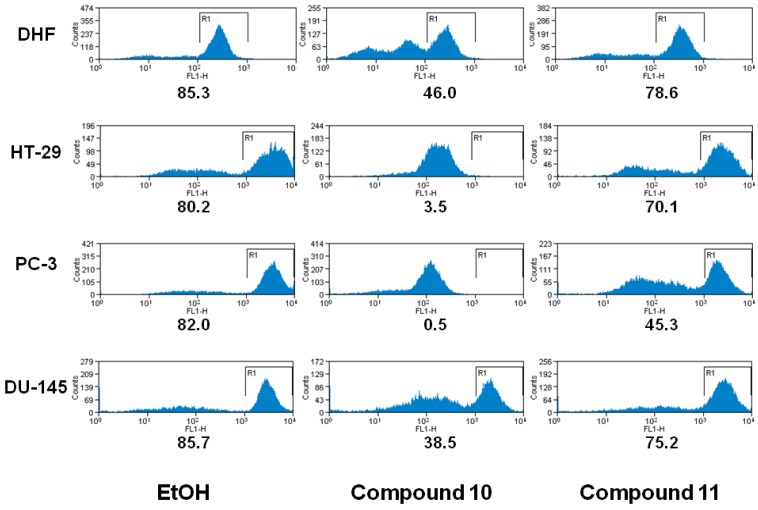
*ent*-Cyclozonarone [(+)-**10**] and **11** treatment-induced changes in the mitochondrial membrane permeability in HDF, HT-29, PC-3 and DU-145 cells. The cells were stained with rhodamine 123, and then analyzed by flow cytometry. Representative histograms show changes in mitochondrial membrane permeability in DU-145, HT-29, PC-3 and DHF cells. The values indicate the percentages of the rhodamine 123 stained cells treated without or with the compounds (+)-10 or 11 (25μM). (* *p* <0.05 or ** *p* < 0.001 *vs*. control treated cells).

Changes induced in the mitochondrial membrane potential (MMP) have been reported previously to represent a determinant in the execution of cell death [19,20]. The effect of compounds (+)-**10** and **11** on the mitochondrial membrane potential was determined by flow cytometry using rhodamine 123 stain [29]. As shown in Figure 5, the percentage of rhodamine 123 stained-cells were 46.0 ± 3.2%, 3.5 ± 1.1%, 0.5 ± 1.0% and 38.5 ± 3.5% in the DHF, HT-29, PC-3 and DU-145 cells, respectively, after treatment with (+)-**10** (25 μM); and 78.6 ± 5.34%, 70.1 ± 6.42%, 45.3 ± 4.42% and75.2 ± 5.2%, respectively, after treatment with **11,** as compared to 80–85% in the control (ethanol-treated) cells. Figure 5 showed that compounds (+)-**10** and **11** induced increased mitochondrial membrane permeability in cancer cells with a greater effect of (+)-**10** in all cell lines tested. Moreover both compounds have a weaker effect on mitochondrial membrane potential in the fibroblast cell line, DHF. Thus, compounds (+)-**10** and **11** induced loss of mitochondrial membrane potential correlated with increased cell death (see Figure 5).

**Figure 6:**
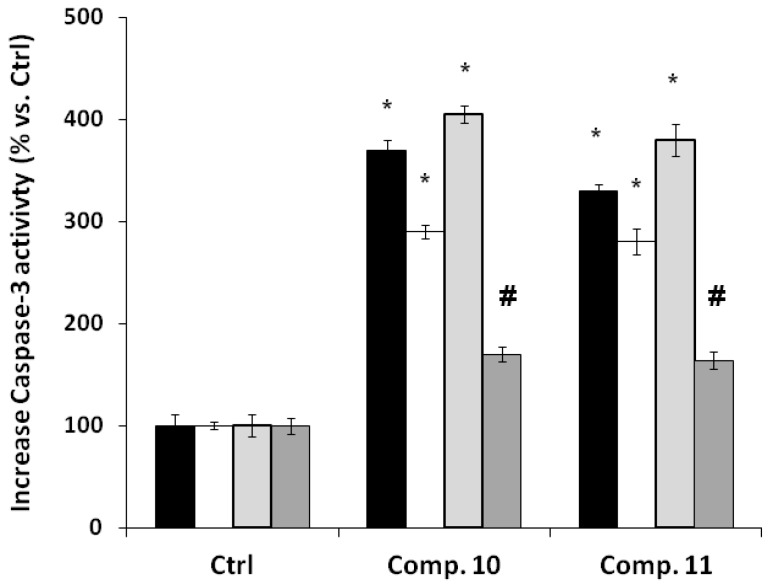
Effect of *ent*-cyclozonarone and compound **11** on caspase-3 activity of DU-145 (black), PC-3 (white), HT-29 (light grey) and DHF (dark grey) cells. Cells were exposed to compounds at 25 µM and incubated for 24 h. Values are mean ± S.D. (*n* = 3). All data are reported as the percentage change in comparison with the vehicle-treated cells, which were arbitrarily assigned 100%. * *p* < 0.05, significantly different from the vehicle-treated cells (1% ethanol in medium, that is, compound concentration = 0) # *p* < 0.001, significantly different from the vehicle-treated cells.

Then we investigated the effects of our compounds on caspase activity. We focused on caspase-3, which is activated by a great number of apoptotic signals. This enzyme is a main executor of apoptosis playing a central role in its biological processing. It has been reported that activation of caspase-3 is an essential event for the induction of oligonucleosomal DNA fragmentation [30]. We analyzed the effect of treatment with the (+)-**10** and **11** on caspase-3 activation in normal and cancer cells. As shown in Figure 6, the activation of caspase-3 in cells exposed to compounds (+)-**10** and **11** is increased versus control-treated cells. (+)-**10** increased the activity of caspase 3 when compared with control by 3.6 ± 0.3, 3.0 ± 0.1, 4.0 ± 0.3, and 1.6 ± 0.2 times in DU-145(black), PC-3 (white), HT29 (light gray) and DHF (dark gray) cells, respectively. Compound **11** increased the activity of caspase 3 by 3.4 ± 0.2, 2.8 ± 0.3, 3.8 ± 0.4, 1.5 ± 0.2 times *versus* control cells in DU-145, PC-3, HT29 and DHF cells, respectively).

## 3. Experimental

### 3.1. General

Unless otherwise stated, all chemical reagents purchased (from Merck or Aldrich) were of the highest purity commercially available and were used without previous purification. Melting points were determined (in triplicate) on a Stuart-Scientific SMP3 apparatus and are uncorrected. Optical rotations were measured with a sodium lamp (λ = 589 nm, D line) on a Perkin Elmer 241 digital polarimeter equipped with 1 dm cells at the temperature indicated in each case. Elemental analyses were obtained in a Fison Instruments EA 1108 microanalyzer.^1^H-, ^13^C- (DEPT 135 and DEPT 90), HSQC and HMBC spectra were recorded in CDCl_3_ solutions and are referenced to the residual peaks of CHCl_3_ at *δ* 7.26 ppm and *δ* 77.0 ppm for ^1^H and ^13^C, respectively, on a Bruker Avance 400 Digital NMR spectrometer, operating at 400.1 MHz for ^1^H and 100.6MHz for ^13^C. Chemical shifts are reported in *δ* ppm and coupling constants (*J*) are given in Hz. Silica gel (Merck 200–300 mesh) was used for column chromatography and silica gel plates HF-254 for TLC. TLC spots were detected by heating after spraying with 25% H_2_SO_4_ in H_2_O. Polygodial (**14**) was obtained by multigram preparative column chromatography (hexane: ethyl acetate gradient) from the crude hexane extract of the ground stem bark of *Drimyswinteri* Forst., as previously described [26]. Compounds **15**–**19** and **13** were synthesized using protocols reported in the literatura and the identities were established by comparison with the previously reported spectral data [26,27,28]. The syntheses of compounds **11**, **12** and **20** are described below.

*(1S,4aS,8aS)-5,5,8a-Trimethyl-1-vinyldecahydronaphthalen-1-ol* (**20**). Ketone **13** (140 mg, 0.72 mmol) was dissolved in anhydrous THF (10 mL) and cooled to −78 °C. Vinylmagnesium bromide (1 mL, of a 1.0 M solution, 1.0 mmol) was added dropwise. The mixture was allowed to warm to room temperature over 1 h, and a saturated aqueous solution of NH_4_Cl was added. The resulting slurry was diluted with ethyl acetate (100 mL) and washed with water (50 mL). The organic phase was dried (MgSO_4_) and concentrated. After a column chromatographic purification using a mixture of hexane and ethyl-acetate with increasing polarity (49:1 and 1:49) alcohol **20** (70 mg, 27%) was obtained as a colourless oil. ^1^H-NMR (CDCl_3_) δ: 6.03 (1H, dd, *J_trans_* = 17.3 Hz; *J_cis_* = 10.9 Hz, vinyl proton), 5.18 (1H, dd, *J_cis_* = 10.9 Hz; *J_gem_* = 0.7 Hz, vinyl proton), 5.09 (1H, dd, *J_trans_* =17.3 Hz; *J_gem_* = 0.7 Hz, vinyl proton), 0.97 (3H, s, 8-α-Me), 0.89 (3H, s, 5-α-Me), 0.84 (3H, s, 5-β-Me). ^13^C-NMR (CDCl_3_) δ: 142.8 (=CH), 113.3 (=CH_2_), 53.5 (C-1), 45.7 (C-4a), 41.9 (C-6), 41.1(C-8a), 33.7 (5-α-Me), 33.4(C-2), 33.2 (C-5), 32.1 (C-8), 21.9 (5-β-Me), 21.9 (C-3), 21.4 (C-4), 18.5 ( C-7), 16.9 (8a-Me),in accordance with literature [28].

*(4αS, 8αS)-4,4,8α-Trimethyl-8-vinyl-1,2,3,4,4a,5,6,8a-octahydronaphthalene* (**12**). Thionyl chloride (0.10 mL, 1.16 mmol) was added dropwise at −30 °C to a solution of enol**20** (70 mg, 0.31 mmol) in anhydrous pyridine (4 mL). The solution was brought to 0 °C and stirred for 1 h. The reaction mixture was diluted with hexane and washed with dilute HCl and then with saturated NaHCO_3_. The organic phase was dried and concentrated. Purification by column chromatography using a mixture of hexane and ethyl-acetate with increasing polarity (49:1 and 1:49) afforded diene **12** (40 mg, 52.5%) as a colorless oil. ^1^H-NMR (CDCl_3_) δ: 6.31 (1H, dd, *J*_cis_ = 10.7 Hz, *J*_trans_ 17.0 Hz, =CH), 5.63 (1H, t, *J* = 3.9 Hz, H-7), 5.23 (1H, dd, *J*_gem_ = 2.2 Hz, *J*_trans_ 17.0 Hz, =CH_2_), 4.89 (1H, dd, *J*_gem_ = 2.2 Hz, *J*_cis_ = 10.7 Hz, =CH_2_), 2.12–0.95 (11H, m), 1.06 (3H, s, 8-α-Me), 0.90 (3H, s, 4-α-Me), 0.85 (3H, s, 4-β-Me),in accordance with literature[28].

*(6aS,10aS)-7,7,10a-Trimethyl-5,6,6a,7,8,9,10,10a-octahydrochrysene-1,4-dione* (**11**). A mixture of diene **12** (40 mg, 0.20 mmol) and *p*-benzoquinone (32 mg, 0.30 mmol) in benzene (2 mL) was stirred at refluxfor 12 h, after which TLC analysis showed the disappearance of the starting material. The solution was allowed to cool to room temperature, and DBU (2 drops) was added with stirring. The reaction mixture was concentrated (rotary evaporator), and the residue purified by column chromatography using a mixture of hexane and ethyl-acetate with increasing polarity (49:1 and 1:49) to yield quinine **11** (5.2 mg, 33.0%) as yellow crystals, m.p. 106–108 °C. [α]${}_{D}^{20}$ = 1,05° (c = 0.3 CHCl_3_). Analysis: calculated for C_21_H_24_O_2_, C = 81.78%, H = 7.84%, found:C = 81.52%, H = 7.77%. The assignment of the signals in the ^1^H-NMR (CDCl_3_) and ^13^C-NMR (CDCl_3_) spectra are shown in the Table 2.

### 3.2. Cell Lines

The experimental cell cultures were obtained from the American Type Culture Collection (Rockville, MD, USA). HT-29 cells (colon cancer cell line), PC-3 and DU-145 (prostate cancer cell lines) and human dermal fibroblasts (DHF) were grown in Dulbecco’s modified Eagle’s medium (DMEM) containing 10% FCS, 100 U/mL penicillin, 100 µg/mL streptomycin and 1 mM glutamine. Cells were seeded into 96 well microtiter plates in 100 µL at a plating density of 5 × 10^3^ cells/well. After 24 h incubation at 37 °C under a humidified 5% CO_2_ atmosphere to allow cell attachment, the cells were treated with different concentrations of drugs and incubated for 72 h under the same conditions. Stock solutions of compounds were prepared in ethanol and the final concentration of this solvent was kept constant at 1%. Control cultures received 1% ethanol alone.

### 3.3. Cell Viability

#### 3.3.1. *In Vitro* Growth Inhibition Assay

The sulforhodamine B assay was used according to the method of Skehan*et al.* [29]. Briefly, the cells were set up at 3 × 10^3^ cells per well of a 96-flat-bottomed, 200 μL well microplate. Cells were incubated at 37 °C in a humidified 5% CO_2_/95% air mixture and treated with the compounds at different concentrations for 72 h. At the end of drug exposure, cells were fixed with 50% trichloroacetic acid at 4 °C. After washing with water, cells were stained with 0.1% sulforhodamine B (Sigma-Aldrich, St. Louis, MO, USA), dissolved in 1% acetic acid (50 µL/well) for 30 min, and subsequently washed with 1% acetic acid to remove unbound stain. Protein-bound stain was solubilized with 100 µL of 10 mM unbuffered Tris base, and the cell density was determined using a fluorescence plate reader (wavelength 540 nm). Values shown are the mean ± SD of three independent experiments in triplicate. The software used to calculate the IC_50_ values was GraphPad (GraphPad Software, San Diego, CA, USA).

### 3.4. Morphological Assessment of Cell Apoptosis

#### Hoechst 33342

Morphological changes in the nuclear chromatin of cells undergoing apoptosis were revealed by a nuclear fluorescent dye, Hoechst 33342. Briefly, on 24-well chamber slides, 1 × 10^4^ cells/mL DHF, HT-29, PC-3 and DU-145 were cultured and exposed to compounds for 24 h. The control group was also exposed to 1% ethanol. The cells were washed twice with phosphate buffer solution, fixed with 3.7% formaldehyde and washed again with phosphate buffer solution. Following the addition of 1µM Hoechst 33342 (Sigma-Aldrich, Santiago, Chile), they were reacted in a dark room at room temperature for 30 min. After washing, they were examined under an immunofluorescence microscope (Olympus IX 81 model inverted microscope).

### 3.5. Analysis of Mitochondrial Membrane Permeability

Rhodamine 123, a cationic voltage-sensitive probe that reversibly accumulates in mitochondria, was used to detect changes in transmembrane mitochondrial membrane potential. Exponentially growing cells were incubated with the compound as indicated in the figure legends. Cells were labelled with 1 μM rhodamine 123 at 37 °C in cell medium for 60 min before terminating the experiment. Cells were detached from the plate, after washing with ice cold PBS, and the samples were analyzed by flow cytometry. Data are expressed in percentage of cells withrhodamine 123 [29].

### 3.6. Caspase 3 Activity Assay

Caspase activity was measured using a colorimetric assay. Briefly, the cells exposed to compounds were collected by centrifugation at 1000 rpm and the cells were lysed with lysis buffer (1% Triton X-100, 0.32M sucrose, 5 mM EDTA, 10 mM Tris–HCl, pH 8.0, 2mM dithiothreitol, 1mM PMSF, 1 g/mL aprotinin, 1 mg/mL leupeptin). Thereafter, the lysates were transferred to wells in a 96-well microplate and were incubated with DEVD-pNA (final concentration 200 µM) specific for caspase-3, at 37 °C for 1 h. The intensity of the developed colour was read at 405 nm in a microplate reader (SpectraMax, Winooski, VT, USA). The results are expressed as percentages of the control level.

## 4. Conclusions

In summary, we describe here the synthesis of a new *ent*-cyclozonarone angular isomer **11** andcompare its cytotoxicity and that of *ent*-cyclozonarone [(+)-**10**] toward normal and cancer human cells. The analyzed compounds induce cell death in different cancer cell lines by inducing dissipation of the mitochondrial membrane potential and the sequential activation of caspase-3 leading to apoptosis, the effects of (+)-**10** being stronger than those of **11**. These compounds displayed promising activity and could be used as leads in the design and development of new anticancer drugs. However, additional studies to determine its *in vivo* biological activities and to identify the anti-tumor potential of these compounds, which may represent a promising candidate for *in vivo* studies of anti-tumor therapies, will be needed.

## Figures and Tables

*Reagents and Conditions*: (a) NaBH_4_/MeOH, 90%; (b) ClTBDPS, imidazole, DMF, 97%; (c) Jones reagent, acetone, 49.4%; (d) TBAF, THF,95%; (e) H_2_, PtO_2_, 79%; (f) Jones reagent, acetone, 60%; (g) vinylmagnesium bromide, THF, 27%; (h) thionyl chloride, Py, 52.5%; (i) *p*-benzoquinone, Bz, reflux, 12 h; (j) DBU, Bz, 2 h, 33%.

**Table 1:** Cytotoxicity (IC_50_ µM) of (+)-**10** and **11**.

Compound	DU-145	PC-3	HT-29	DHF
(+)-**10**	6.4 ± 0.8	9.5 ± 1.2	25.0 ± 0.9	48.0 ± 2.3
**11**	13.3 ± 1.4	14.9 ± 1.8	45.0 ± 0.5	91.0 ± 7.8

**Table 2:** ^1^H-NMRchemical shifts, δ (ppm), coupling constants J (H,H) (Hz), and ^13^C-NMR chemical shifts, δ (ppm), for compound **11**.

Position	^1^H-NMR	^13^C-NMR
1	__	185.6
2	6.87 (1H, s)	141.1 *
3	6.87 (1H, s)	136.2 *
4	__	187.7
4a	__	129.2
4b	__	139.9
5	3.52 (1H, ddd, 6.0, 6.0, 19.7); 3.29 (1H, ddd, 8.1, 11.2, 19.7)	30.2
6	1.69 (2H, m)	19.3
6a	1.34 (1H, dd, 2.01, 12.4)	49.0
7	__	29.7
8	1.53 (1H, bs, 13.2); 1.3 (1H, m)	41.2
9	2.02 (1H, m); 1.65 (1H, m)	18.8
10	2.34 (1H, bs, 12.7); 1.41 (1H, dd, 3.7, 12.9)	39.2
10a	__	33.4
10b	__	158.3
11	7.72 (1H, d, 8.3)	130.2
12	7.98 (1H, d, 8.3)	124.9
12a	__	131.3
7-α-Me	1.00 (3H, s)	33.1
7-β-Me	0.97 (3H, s)	24.7
10a-Me	1.27 (3H, s)	21.6

* exchangeable signals.
